# P-2031. Implementation of a Quality Improvement Model for Evaluation of Community-acquired Pneumonia Management in the Coccidioidal Endemic Region

**DOI:** 10.1093/ofid/ofaf695.2195

**Published:** 2026-01-11

**Authors:** Justin F Hayes, Neil M Ampel

**Affiliations:** University of Arizona, Tucson, AZ; University of Arizona, Tucson, AZ

## Abstract

**Background:**

Coccidioidomycosis (CM) is an infection caused by the fungal pathogens *Coccidioides* and is endemic in the southwestern United States. It is estimated that approximately 25% of cases of community-acquired pneumonia (CAP) in this region are due to *Coccidioides*, presenting unique challenges to antimicrobial stewardship programs there. The objective of this project was to establish a continuous quality improvement model to collect information regarding CAP management practices at a major medical center in Arizona.

**Methods:**

A daily reporting system was established to identify all adult patients admitted to Banner University Medical Center-Tucson that were coded for pneumonia with ICD-10 code J18.9 from December 1, 2024 – April 18, 2025. Patient demographic information as well as CM serologic testing and antimicrobial prescription patterns were collected.

**Results:**

225 patients were included for analysis. 56% (126/225) patients had CM serologic testing performed with 71.4% (40/56) patients having CM testing performed in the intensive care unit. Monthly trends demonstrated variable rates of CM testing and CM positivity. Of the 126 patients who had CM testing performed, 9 (7.1%) had positive results, all being returned while hospitalized. Two patients were started on antifungals, while all 9 patients received a full course of antibacterials for CAP. Only 67.1% (151/225) of all patients received CAP-guideline recommended antibiotic therapy.

**Conclusion:**

The results demonstrated that only 56% of patients hospitalized with CAP at a major medical center in Arizona were tested for CM, as recommended by the CDC, and those diagnosed continued to receive antibacterials. These results affirm the importance of establishing a CAP continuous quality improvement program in the coccidioidal endemic region focused on CM testing and antimicrobial stewardship.

**Disclosures:**

All Authors: No reported disclosures

